# Effect of *Spirulina platensis* on the content values of wheat bread

**DOI:** 10.1038/s41598-026-43788-y

**Published:** 2026-03-23

**Authors:** Georgina Takács, Beatrix Sik, Rita Székelyhidi

**Affiliations:** Department of Food Science, Albert Kázmér Faculty of Agricultural and Food Sciences, Széchenyi István University, 15-17 Lucsony Street, Mosonmagyaróvár, 9200 Hungary

**Keywords:** Spirulina, Bread, Health benefits, Content values, Polyphenols, Antioxidant activity, Biochemistry, Biotechnology, Microbiology, Plant sciences

## Abstract

Due to their nutritional composition, algae are promising ingredients in the development of new foods. The aim of our work was to prepare bread containing *Spirulina platensis* (new name *Arthrospira platensis*) in different percentages (0.5, 1.0, 2.5%) within the framework of the MSZ 6369/8-1988 standard, and to determine its content (dry matter, ash, fat, protein/nitrogen, fiber content, carbohydrate content, and polyoxide, color), as well as its texture and color. Furthermore, we assessed consumer opinions through a sensory evaluation. We found that increasing the width and shape fraction while decreasing the height. The results showed that the antioxidant and polyphenols properties of Spirulina-enriched breads increased. The protein, nitrogen, fibre content increased and carbohydrate, energy value properties of Spirulina-enriched breads decreased with increasing concentration of algae. Spirulina powder increased the greenness of the bread and decreased the lightness of the crumb. The hardness, cohesiveness and springiness increased with the addition of Spirulina to bread, while the gumminess and chewiness values became lower compared to the control. Consumer acceptability results showed that the addition of Spirulina at a concentration of 2.5% significantly reduced overall acceptance. Our results indicated that *Spirulina* cyanobacteria, can be a suitable raw material for making bread, also from the point of view of healthier and sustainable nutrition.

## Introduction

Spirulina, also known as blue-green algae-cyanobacteria, is a prokaryotic microorganism that gets its name from its spiral shape^1^. Spirulina, also known as Arthrospira, has been used by humans for a very long time as a food or dietary supplement^2^. The Spirulina species most commonly used as a dietary supplement are *Spirulina platensis* and *Spirulina maxima*^3^. Nowadays, Spirulina can also be found on the shelves of organic stores in the form of powder, tablets and various drinks as a dietary supplement^2^. The WHO refers to it as a superfood, as it functions as a complete food thanks to its rich nutrient content^4^. *Arthrospira platensis* is an extremely rich source of protein (55-70%, w/w), and also contains 47% essential amino acids such as methionine, which is usually absent in other algae and cyanobacteria. It contains 15-25% carbohydrates, 8-13% minerals, 3-7% fats, 8-10% fibers and 6-7% moisture^5^. The phrase “eat healthy and live healthy” is becoming increasingly popular in our society as a fundamental requirement for a long life^6^. Today, people not only expect food to meet their energy and nutritional needs, but also expect certain foods to provide health benefits that may even help prevent non-communicable diseases, such as cardiovascular diseases, by reducing risk factors for these diseases^7^. Wheat is one of the most widely grown cereals and plays a significant role in Hungary’s food supply. In Europe, approximately 56% of daily protein intake comes from animal sources (meat, milk, fish), while plant-based proteins account for around 43%^8^. More than half of the latter consists of wheat protein, making wheat and other cereals such as corn and rice a staple. Given the growing global population, identifying and evaluating alternative (non-animal) protein sources has become increasingly important.

In recent years, consumer awareness of health benefits has been a key factor in food choices, including an increased interest in macro- and microalgae. Although seaweed has traditionally been a part of Eastern diets, its consumption has risen in Western countries due to globalization and its recognized health benefits^9^. The European Commission has also promoted increased algae consumption as a sustainable alternative protein source^10^.

Spirulina consumption offers various health benefits, including anti-inflammatory, antimicrobial, antihypertensive, and anticoagulant effects and support for weight management and disease prevention^11,12^. Additionally, the inclusion of algae enhances the perception of saltiness, allowing for a reduction in the product’s overall salt content^13^. Microalgae can also influence various food properties, such as texture, color, solubility, foaming ability, water and oil absorption, and emulsification of other ingredients^14^.

This study aimed to prepare bread incorporating *Spirulina platensis* at different concentrations (0.5%, 1.0%, and 2.5%) following the MSZ 6369/8-1988 baking standard. The composition of the bread was analyzed, including dry matter, ash, fat, protein/nitrogen, fiber, carbohydrate content, and energy value. Its texture, color, antioxidant activity, and polyphenol content were also examined. A sensory evaluation was conducted to assess consumer perception of the Spirulina-enriched bread. This study addresses the following question: How does the use of the cyanobacterium *Spirulina platensis* in food affect consumer liking and product physicochemical properties?

## Material and methods

### Baking

Bread containing commercially available *Spirulina platensis* was prepared for a baking test based on the MSZ 6369/8-1988 standard. This standard provides information on the quality of flour obtained from milling bread grains. The test was used to evaluate the suitability of cyanobacteria as a partial flour replacement.

During the baking test, BL 55 wheat flour (commercially available flour) was used as the main ingredient for all breads, and *Spirulina platensis* powder (distributor Paleolcentrum Kft., place of origin China) was added to experimental breads at concentrations of 0.5%, 1.0%, and 2.5%, calculated based on the weight of flour (w/w). Used spirulina powder in 0.5, 1.0 and 2.5% instead of BL 55 wheat flour. The control bread was prepared without *Spirulina platensis*. Following the MSZ 6369/8-1988 standard, the basic bread recipe (control) included flour (100%), yeast (3%), sugar (0.5%), salt (quantity as specified by standard), and water (180 mL per defined flour weight). For Spirulina-enriched breads, Spirulina *platensis* powder was incorporated into the dough mixture at the specified percentages (0.5%, 1.0%, and 2.5%) relative to flour weight, resulting in a proportional reduction of flour content. After mixing ingredients thoroughly, dough was formed into loaves, placed on baking trays, and allowed to rise for 50 minutes at 31°C.

After the initial rise, the loaves were weighed to 400 g, carefully reshaped, and placed back into the thermostat at 31°C for 40 minutes. The relative humidity was maintained at a minimum of 85%. Finally, the risen dough was baked at 260°C in an oven with 93% relative humidity for approximately 25–30 minutes.

### Physicochemical analysis

The physical characteristics of Spirulina-enriched breads were analyzed in terms of weight, width, height, and shape ratio (width/height). Crude protein and nitrogen content were determined using the Dumas method, applying a nitrogen conversion factor of 6.25. Crude ash, fat, and fiber were measured according to the method described by Pauline et al.^15^, along with the determination of dry matter content.

Carbohydrate content was calculated using the formula provided by Rodrigues^16^:

$$Carbohydrate \,content = dry matter\, content - (protein\, content + fat \,content + ash\, content + raw\, fiber\, content)$$

The energy value of the bread was determined with the Merrill et al.^17^ formula:

$$Energy\, value = 4 \times protein\, content + 4 \times carbohydrate\,content + 9 \times lipid \,content$$

### Antioxidant and polyphenol activity measurements

#### Sample preparation

The Spirulina-enriched bread samples were frozen and then ground using a hammer grinder. For extraction, 2 g of ground bread was weighed into Erlenmeyer flasks using an analytical balance, followed by the addition of 20 mL of an extraction mixture containing methanol and water (80:20 V/V%). The extraction process was performed at 65°C in an ultrasonic bath for 1 hour. The resulting extracts were centrifuged at 2500 g for 20 minutes at room temperature, and the filtrate was collected for further analysis.

#### FRAP assay

The antioxidant content of the Spirulina-enriched bread samples was estimated using the method described by Benzie and Strain^18^^,^ as modified by Pulido et al.^19^, with minor adjustments. A total of 200 μL of the extracted sample, 3 mL of ferric reducing antioxidant power (FRAP) solution, and 100 μL of water were pipetted into a test tube. The prepared solutions were stored in a dark environment for 5 minutes, after which their absorbance was measured using a Spectroquant Pharo 100 spectrophotometer (Merck, Germany) at a wavelength of 593 nm against a blank. Ascorbic acid (40–500 mg/L) was used as the standard, and the results were expressed as mg ascorbic acid equivalent capacity (AAE)/g dry matter.

#### Folin–ciocalteu assay

Total polyphenol content was determined based on the Folin–Ciocalteu method described by Singleton et al.^20^^,^ with some modifications^21^. A total of 200 μL of Spirulina-enriched bread extract was pipetted into a test tube along with 1.5 mL of high-purity water. The reagents were added sequentially: 2.5 mL of Folin–Ciocalteu reagent, followed by 2 mL of Na_2_CO_3_. The tubes containing the mixture were kept in a dark environment for 90 minutes, after which the absorbance was measured at 725 nm against a blank. Gallic acid (25–1000 mg/L) was used as the standard.

### Texture profile analysis

Texture characteristics were analyzed using an IMADA FRTS-50N-I texture analyzer (Northbrook, USA) to determine the Spirulina-enriched bread samples’ hardness, cohesiveness, springiness, gumminess, and chewiness. The analysis was conducted using Force Recorder (ZT-RP) software, with the following parameter settings: a compression probe with a 40 mm diameter, a pre-test speed of 2 mm/s, a post-test speed of 10 mm/s, a distance of 2 mm from the table, and a trigger force of 0.05 N.

### Crumb color

A 3 cm thick slice of bread was cut and placed on a white surface. A portable colorimeter (Spectral Colorimeter MC-286, Shenzhen, China) was used to measure the color of the bread. The colorimeter provided L*, a*, and b* values in the CIELAB color space: L* represents lightness, ranging from black (0) to white (100); a* indicates red-green saturation (+a* = red, -a* = green); and b* represents yellow-blue saturation (+b* = yellow, -b* = blue).

### Consumer acceptance

Consumer acceptance of the bread was evaluated at the Department of Food Science, Széchenyi István University (Mosonmagyaróvár). Thirty-three untrained consumers (15 men and 18 women; average age: 24 years) participated in the study. While the panel size was smaller than typically required for a fully representative analysis, it was sufficient to provide preliminary insights into consumer perceptions of Spirulina-enriched breads. Sensory attributes—including external appearance, texture, odor, taste, and color—were rated using a five-point hedonic scale, where 1 indicated strong dislike, 3 corresponded to neither like nor dislike, and 5 indicated strong liking.

### Statistical analysis

The results were expressed as the mean ± standard deviation (SD). A one-way analysis of variance (ANOVA), followed by Tukey’s multiple comparison test, was used to determine significant differences in the data (*p* < 0.05). These methods are particularly well suited to comparing means across multiple groups. All measurements were performed in triplicate.

### Ethical approval

All procedures performed in studies involving human participants were in accordance with the ethical standards of the institutional and/or national research committee. We obtained informed consent from all subjects before the tasting. Hungarian laws, specifically Act CLIV of 1997 on Healthcare, do not explicitly define “exempt research.” However, they outline situations where IRB review is not mandatory.

## Results and discussion

### Microalgae characteristics

The dry material content of *Spirulina platensis* is 93.70±0.00%, with an ash content of 7.02±0.01%, fat content of 7.70±0.01%, protein content of 57.50±0.00%, fiber content of 8.02±0.02%, carbohydrate content of 20.30±0.11%, and an energy value of 388 kcal/100 g. The protein content of microalgae varies depending on the algal species, season, year, environmental conditions, and geographic location^22^. Spirulina microalgae are particularly rich in protein, containing approximately 70% protein by dry weight, making them a valuable food supplement and ingredient for food enrichment. Amoriello et al.^9^ reported similar protein content (63.5 g/100 g dw) in spirulina but observed a lower carbohydrate content (9.1 g/100 g dw).

Edible microalgae generally contain less dietary fiber than macroalgae^22^. For example, sea lettuce has a higher fiber content (34.4 g/100 g dw) compared to spirulina (7.0 g/100 g dw)^9^. Incorporating high-fiber seaweed into food products can help meet the recommended daily fiber intake of 30–35 g/day for men and 25–32 g/day for women—the standard in most Western countries^23^.

### Analysis of spirulina-enriched breads

#### Physical and chemical characteristics

The weight of the breads were similar with the addition of algae. There was a significant increase in the width of Spirulina-enriched bread, from 13.80±0.61 cm (control) to 14.97±0.87 cm (1.0%), while the height decreased to 4.85±0.21 cm (1.0%) compared to the control (5.15±0.35 cm). The addition of 2.5% spirulina (341.64±0.03 g) reduced the weight of the bread compared to the control (343.03±0.04 g) (Table 1). Choton et al.^24^ investigated the effect of adding 3% spirulina powder and different percentages of seabuckthorn on bread. They observed an increase in weight as the seabuckthorn concentration increased, ranging from 145,20 g to 153.70 g, compared to the control (144.00 g). Manitoba flour showed the highest height (37-48 mm) compared to the other samples. The addition of spirulina significantly (p<0.05) affected the bread weight, the biggest weight showed standard bakery flour (47.3 g in 1.5 % spirulina)^25^. The weight of Spirulina-enriched (5, 10, 15%) pan bread increase (75.1-79.0 g) compared to the control (72.2 g)^26^. Montevecchi et al.^27^ measured the weight and height of breads made with different percentages (1 and 2%) of spirulina powder and "Italian type 1" semi-whole wheat flour. The weight (215.51±0.54 and 216.63±0.23 g) and height (5.06±1.61 and 5.11±1.61 cm) of Spirulina-enriched breads were not significantly greater than the control (215.78±0.68 g,5.14±1.71 cm).

**Table 1:** Physical Characteristics of spirulina-enriched Breads (n = 3).

**Parameters**	**Control**	**0.5 %**	**1.0 %**	**2.5 %**
Weight (g)	343.03±0.04^a^	341.20±0.05^a^	342.78±0.05^a^	341.64±0.03^a^
Width (cm)	13.80±0.61^d^	14.17±0.15^c^	14.97±0.87^a^	14.47±0.15^b,c^
Height (cm)	5.15±0.35^b^	5.50±0.14^a^	4.85±0.21^d^	5.00±0.42^c^
Shape fraction (W/H)	2.72±0.34^c^	2.59±0.05^d^	3.19±0.21^a^	2.92±0.23^b^

Different letters (a, b, c, d) indicate significant differences in the rows (*p* ≤ 0.05).

Table 2 shows the biochemical composition of Spirulina-enriched breads, with a significant increase in protein at 1.0% and 2.5% (12.52±0.07 % and 13.51±0.04 %, respectively) compared to the control. In comparison, crude fiber was significantly higher in the 2.5% Spirulina-enriched bread (1.40±0.07 %) than the control (1.10±0.00%). Rojo-Poveda^28^ described that a higher fiber content can affect protein content because fibers absorb some of the water, making it unavailable for the development of the gluten network. Furthermore, higher fiber content can cause a break in the protein structure, affecting the dough’s expansion ability.

**Table 2:** Biochemical Composition of Spirulina-enriched Breads (n = 3).

**Parameters (%)**	**Control**	**0.5 %**	**1.0 %**	**2.5 %**
**Dry material**	66.90±0.06^a^	66.20±0.04^b^	66.06±0.07^c^	66.02±0.02^c^
**Crude ash**	3.68±0.03^b^	3.66±0.08^c^	3.65±0.05^c^	3.72±0.03^a^
**Protein**	11.99±0.09^a^	12.22±0.07^b^	12.52±0.07^c^	13.51±0.04^d^
**Nitrogen**	1.92±0.02^d^	1.95±0.01^c^	2.00±0.02^b^	2.16±0.01^a^
**Crude fiber**	1.10±0.00^a^	1.11±0.00^a^	1.28±0.07^b^	1.40±0.07^c^
**Carbohydrates**	50.13±0.13^a^	49.21±0.13^b^	48.96±0.16^c^	47.26±0.43^d^
**Energy value (Kcal/100 g)**	256.13±0.34^a^	253.55±0.42^b^	253.91±0.31^c^	251.72±0.86^d^

Different letters (a, b, c, d) indicate significant differences in the rows (*p* ≤ 0.05).

The dry material content decreased significantly (66.02–66.20%) compared to the control (66.90%). The ash content was significantly higher in bread enriched with 2.5% spirulina (3.72%), while it was significantly lower in bread enriched with 0.5% and 1.0% spirulina (3.66–3.65%) compared to the control (3.68%). The fat content in the control and all three Spirulina-enriched breads was 0.00%. The carbohydrate content decreased significantly with increasing spirulina addition. Overall, the 0.5% and 1.0% spirulina samples (253.55 and 253.91 kcal) had lower energy values, while the 2.5% spirulina sample (251.72 kcal) showed a significantly lower energy value compared to the control bread (256.13 kcal).

The fat, protein, fibre, ashcontent of bread increased significantly in all the treatments. The carbohydrate content was higher at higher seabuckthorn concentrations (52.80-55.64 %), compared to the control (51.92 %)^24^. Hernández-López et al^25^ used 4 types of wheat flour and 1.5 and 2.5% Spirulina concentrations. The protein content of all flour types increased with the addition of spirulina compared to the control, but not significantly (e.g. 10.5-11.1% for whole wheat flour,10.3% for control).

Similar results were reported by Hussein et al.^26^ for pan bread made from quinoa flour with the addition of 5, 10 and 15 % spirulina. Protein, fat and ash contents were significantly higher, while fiber content was significantly increased only with the addition of 15 % spirulina. Carbohydrate content was significantly reduced for all concentrations (64.73±1.05-58.54±1.16 %) compared to the control (67.38±1.43 %). The ash content of the breads was significantly higher only in the bread enriched with 2% spirulina (3.24±0.05 g/100 g dry weight) compared to the control (2.57±0.05 g/100 g dry weight) Montevecchi et al.^27^.

#### Antioxidant and polyphenol content

The results of the antioxidant content obtained by the FRAP assay for the examined Spirulina-enriched bread varieties are shown in Table 3. The highest antioxidant content was observed in the 2.5% spirulina bread (0.74 mg AAE/g) compared to the control (0.40 mg AAE/g), with significant differences in each case. Amoriello et al.^9^ reported a higher antioxidant content in bread enriched with 2.5% and 4% spirulina (0.41–0.48 µg TE/mg dw) compared to the control (0.37 µg TE/mg dw). The antioxidant capacity of the breads (1.5 and 2.5 % spirulina) ranged from 400 to 1200 mg EAA/100 g dry weight^25^.

**Table 3:** Total Antioxidant Capacity (TAC) and Total Polyphenol Content (TPC) of Spirulina-enriched Breads (n = 3).

**Parameters**	**Control**	**0.5 %**	**1.0 %**	**2.5 %**
TAC (mg AAE/g)	0.40±0.01^d^	0.45±0.00^b^	0.44±0.01^c^	0.74±0.01^a^
TPC (mg GAE/g)	0.69±0.07^c^	0.70±0.01^b^	0.74±0.02^a^	0.62±0.05^d^

Different letters (a, b, c, d) indicate significant differences in the rows (*p* ≤ 0.05).

The results of the total polyphenol content were obtained using the Folin–Ciocalteu assay. The highest polyphenol content was observed in the 1.0% Spirulina-enriched bread (0.74 mg GAE/g) compared to the control (0.69 mg GAE/g). However, the polyphenol content was significantly reduced in bread enriched with 2.5% spirulina (0.62 mg GAE/g). The addition of 2.5% spirulina significantly increased the TPC in breads made from Manitoba and standard bakery flour, corresponding to 60.2 ± 4.0 mg GAE/100 g dry weight^25^. Nunes et al.^29^ reported that the addition of 1% fresh *C. vulgaris* resulted in an increase in the TPC of bread, while the TPC of commercially available *C. vulgaris* was the same as the control.

Several studies have observed an increase in polyphenols and antioxidant capacity after the incorporation of microalgae in pasta^30^ or wheat flour tortillas^31^, demonstrating the potential of microalgae to increase the antioxidant capacity of foods. Fratelli et al.^32^ also reported an increase in antioxidant and polyphenol content when 3% spirulina was added (TPC 51.70±0.00 mg GAE,7.49±0.10 µM TE/g) compared to the control (TPC 27.70±0.00 mg GAE,2.06±0.02 µM TE/g).

#### Texture profile analysis

Based on Table 4, the average force required to compress the Spirulina-enriched breads significantly increased in the experimental samples (1.58–6.59 N/m^2^) compared to the control (1.34 N/m^2^). Hardness was examined after 0, 24 and 48 hours^32^ and the spirulina-enriched bread had a reduced hardness (2.12±0.43-4.17±0.47 N) compared to the control (1.97±0.26-4.39±0.71 N). Hussein et al^26^ found that the hardness of pan bread increased with increasing spirulina concentration (525-550 N) compared to the control (516 N). *Spirulina platensis* significantly increases the hardness of bread, more than proteins. Increasing the concentration of Spirulina significantly increased the protein content of the baguette^33^. The protein content of Spirulina is about 64-74%, which can lead to excessive crosslinking, making the dough stiff and making it difficult for gas cells to expand^34^. Higher protein levels have been shown to increase bread hardness^35^. Protein content also affects elastic properties such as gumminess, springiness, and chewiness^36^. Others have reported a decrease in breads enriched with 10 g of spirulina powder prepared in three ways compared to the control^37^.

**Table 4:** Texture Profile Analysis (TPA) of Spirulina-enriched Bread (n = 3).

**Parameters**	**Control**	**0.5 %**	**1.0 %**	**2.5 %**
Hardness (N/m^2^)	1.34±0.25^d^	1.80±1.22^b^	1.58±0.23^c^	6.59±1.75^a^
Cohesiveness (J/m^3^)	0.561±0.03^c^	0.578±0.10^b^	0.618±0.03^a^	0.587±0.04^b^
Springiness (N/m^2^)	0.753±0.02^c^	0.784±0.09^a^	0.714±0.06^d^	0.764±0.04^b^
Gumminess (N/m^2^)	7.50±1.09^a^	5.10±1.10^d^	6.30±1.51^b^	5.83±0.89^c^
Chewiness (N/m^2^)	5.64±0.81^b^	4.14±0.69^d^	6.91±0.66^a^	4.33±0.45^c^

Different letters (a, b) indicate significant differences in the rows (*p* ≤ 0.05).

Similar changes were observed for cohesiveness (0.578–0.618 J/m^3^) compared to the control (0.561 J/m^3^). The highest cohesiveness was achieved with the methanol extract of spirulina, while the lowest cohesiveness was observed with the ethanol extract, which showed no significant difference compared to treatments containing spirulina powder^37^. Spirulina also has a significant effect on cohesiveness. Low cohesiveness means an increase in the tendency of bread to crumble^38^. Protein is the main parameter that influences the rheological properties of bread. Increasing the protein concentration reduces the cohesiveness of bread. Microalgae, due to their different protein composition, negatively affect cohesiveness, contributing to a higher protein content, so the cohesiveness of the bread texture is expected to decrease^39^.

Springiness was significantly higher in the experimental samples (0.784 N/m^2^ at 0.5% and 0.764 N/m^2^ at 2.5%) than the control bread (0.753 N/m^2^). Likewise, chewiness was higher in the 1.0% Spirulina-enriched bread (6.91 N/m^2^) compared to the control (5.64 N/m^2^). The highest springiness was observed for spirulina powder (10.56 mm), which was not significantly different from the ethanol extract of spirulina powder (10.53 mm). The lowest springiness was observed for the control (8.43 mm)^37^.

Gumminess significantly decreased in the experimental samples (5.10–6.30 N/m^2^) compared to the control (7.50 N/m^2^). The addition of spirulina powder resulted in the highest gumminess (2.23 N). Ethanol (1.24 nm) and methanol (1.13 nm) extracts did not show significant differences in gumminess. The lowest gumminess was also observed in the control (0.84 N)^37^.

#### Color measurement

Color analysis showed differences in lightness (L*) and color trends (a*, b*) of the products compared to the control (Table 5). Among the breads, the bread enriched with 2.5% spirulina was darker (L* 52.75±1.25) compared to the control (L* 85.51±0.35). As the amount of spirulina increased, the negative a* values (indicating green color) became significantly more pronounced (-2.09 at 0.5%; -2.66 at 2.5%) compared to the control (-0.96), showing a greener hue. Likewise, the b* values, representing yellow hue, increased significantly from 18.19 in the control to 22.44 at the highest spirulina level. The lightness of the samples containing seabuckthorn and spirulina powder (74.40-75.29) were lower compared to control sample (75.40)^24^, we also observed similar values. Spirulina powder which changed the colour of the bread to a darker tone. In contrast, the redness (a*) and yellowness (b*) of the samples presented higher values (1.72-2.15 and 19.77-21.05) compared to the control (1.70 and 20.01), we also observed similar values.

**Table 5:** Color Measurement of Spirulina-enriched Bread (n = 3).

**Parameters**	**Control**	**0.5 %**	**1.0 %**	**2.5 %**
L^*^	85.51±0.35^a^	65.10±1.12^b^	64.37±1.23^c^	52.75±1.25^d^
a*	-0.96±0.13^d^	-2.09±0.15^c^	-2.49±0.13^b^	-2.66±0.14^a^
b^*^	18.19±0.22^d^	21.26±0.29^c^	22.01±0.26^b^	22.44±0.24^a^

Different letters (a, b) indicate significant differences in the rows (*p* ≤ 0.05).

The addition of spirulina significantly affected the a* and b* values, which were lower in all breads containing spirulina, as expected due to the green color of the microalgae powder. The crumbs of the different breads visually displayed a greenish color, the intensity of which increased with higher microalgae dosage levels^25^. Similar values were observed by Fratelli et al.^32^ in bread enriched with spirulina biomass 3 % (L*: 36.04±0.73,a*: -2.24±0.27,b*: 22.39±0.29) compared to the control (L*: 63.26±1.72; a*: -0.62±0.02; b*: 17.60±0.57). Hussein et al^26^ reported a decrease in lightness, redness, and yellowness with increasing spirulina concentration (5, 10, 15 %) in pan bread.

#### Consumer acceptance

**Fig. 1:**
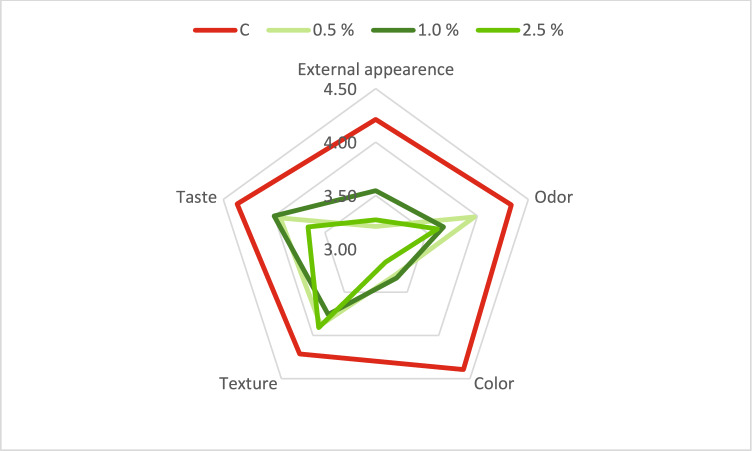
Consumer acceptance evaluation of spirulina-enriched breads C: Control sample; 0.5%, 1.0%, and 2.5% Spirulina-enriched breads.

The consumer acceptability results, shown in Figure 1, revealed a decrease in external appearance scores for Spirulina-enriched breads (0.5%: 3.21; 1.0%: 3.55; and 2.5%: 3.27) compared to the control sample (4.21). Sensory scores, particularly for taste, declined, especially for bread enriched with 2.5% spirulina (3.67), likely due to undesirable side flavors associated with algal ingredients. Hernández-López et al.^25^ the 2.5% concentration samples were generally well accepted by consumers, highlighting the salty taste as a pleasant characteristic. No significant sensory differences were observed among the samples among the 4 flour types and concentrations.

Tasters reported negligible differences in the odor properties of Spirulina-enriched bread. In terms of color, the bread enriched with 2.5% spirulina (3.15) was the least liked by tasters. Sanjari et al.^37^ during sensory evaluation, the control samples received the highest scores for all sensory attributes. The samples enriched with spirulina powder received the lowest scores, based on the judges’ opinions, the green color of the bread was not liked, although some considered this color to be innovative and novel. The color, flavor and texture scores of the breads enriched with ethanol, methanol extract and spirulina powder decreased significantly compared to the control sample, yet the breads containing ethanol and methanol extracts of spirulina received better scores than the breads containing spirulina powder.

Many studies have shown that increasing the amount of seaweed/microalgae tends to decrease consumer acceptance^40^.

Jönsson et al.^41^ studied consumer perception and found it to be influenced by three factors:

- The different grain size, which affects the texture of the bread.
- The taste of seaweed/microalgae.
- The green color, which many consumers associate with mold.

## Conclusion

The incorporation of spirulina powder significantly affected the bread’s organoleptic acceptability, texture, and physical and chemical characteristics. We found an increase in width and shape ratio, while height decreased. The results showed that the antioxidant and polyphenol properties of Spirulina-enriched breads increased.

Protein, nitrogen, and fiber content increased, while carbohydrate and energy values decreased with higher spirulina concentration (2.5%).

Increasing the percentage of spirulina powder increased the bread’s greenness and yellow hue while reducing the crumb’s lightness. Hardness, cohesiveness, and springiness increased with the addition of spirulina, while gumminess and chewiness values were lower than in the control. The consumer acceptability results showed that adding *Spirulina platensis* at a concentration of 2.5% significantly reduced overall acceptance.

Our results indicate that spirulina algae can be a good ingredient for bread making, contributing to healthier and more sustainable diets. However, we acknowledge the limitations of this study, including the small sensory panel size. Future research should explore culinary techniques, such as mixing spirulina with spices or other flavoring ingredients, to improve palatability and consumer acceptance. In addition, further studies could investigate its concentrations to maximize nutritional benefits while maintaining desirable sensory properties.

## Data Availability

The data that support the findings of this study are available on request from the corresponding author.
